# Mental representation of autobiographical memories along the sagittal mental timeline: Evidence from spatiotemporal interference

**DOI:** 10.3758/s13423-021-01906-z

**Published:** 2021-03-29

**Authors:** Alice Teghil, Isabel Beatrice Marc, Maddalena Boccia

**Affiliations:** 1 Department of Psychology, “Sapienza” University of Rome, Rome, Italy; 2 Cognitive and Motor Rehabilitation and Neuroimaging Unit, IRCCS Fondazione Santa Lucia, Rome, Italy

**Keywords:** Semantic memory, Episodic memory, Mental timeline, Mental time travel, Spatiotemporal interference

## Abstract

Time is usually conceived of in terms of space: many natural languages refer to time according to a back-to-front axis. Indeed, whereas the past is usually conceived to be “behind us”, the future is considered to be “in front of us.” Despite temporal coding is pivotal for the development of autonoetic consciousness, little is known about the organization of autobiographical memories along this axis. Here we developed a spatial compatibility task (SCT) to test the organization of autobiographical memories along the sagittal plane, using spatiotemporal interference. Twenty-one participants were asked to recall both episodic and semantic autobiographical memories (EAM and SAM, respectively) to be used in the SCT. Then, during the SCT, they were asked to decide whether each event occurred before or after the event presented right before, using a response code that could be compatible with the back-to-front axis (future in front) or not (future at back). We found that performance was significantly worse during the non-compatible condition, especially for EAM. The results are discussed in light of the evidence for spatiotemporal encoding of episodic autobiographical memories, taking into account possible mechanisms explaining compatibility effects.

**Supplementary Information:**

The online version contains supplementary material available at 10.3758/s13423-021-01906-z.

## Introduction

Autobiographical memory has been defined as "the ability of people to remember their own lives" (Baddeley, 1990). It is a complex process, involving thoughts, affections, intentions, and ambitions (Rubin, 2003). Within the memory systems, autobiographical memory is placed into the long-term memory system (Squire, 1987, 1992; Squire & Zola-Morgan, 1991; Tulving, 1972, 1983, 1985, 2002a, b), as a component of declarative (or explicit) memory. It can be roughly divided into an episodic (EAM) and a semantic (SAM) component. The former encompasses events with a unique spatiotemporal context. The latter corresponds to memory for information with no clear temporal and spatial context, such as the names of schoolteachers, and the names of family members or friends. SAM does not involve the re-experiencing of past events, whereas EAM entails the recollection of events from a specific time and place, the re-experiencing of contextual details, and the awareness of the self as a continuous entity across time (Levine et al., 2002). According to Tulving (1983), autonoetic consciousness is a defining property of episodic memory that includes “remembering” as well as the experience of mental time travel, in which personal experiences of past events are reinstated. Conversely, noetic consciousness does not involve any self-recollection, and roughly corresponds to the awareness of “knowing.”

The study of autobiographical memory is thus strictly linked to that of the experience of mental time travel. Mental time travel, indeed, crucially depends on the conceptualization of space and time, which allows us to define and encode events according to *where* and *when* they happened (Corballis, 2019), along a continuum. Interestingly, an asymmetric relation seems to exist between space and time: evidence from psycholinguistic and psychophysical experiments (Boroditsky, 2000; Boroditsky & Ramscar, 2002; Núñez & Sweetser, 2006; Piaget, 1969; Torralbo et al., 2006; Tversky et al., 1991) suggests that spatial concepts develop earlier than temporal ones, and are needed to structure the conceptual representation of time. In this view, whereas the representation of space directly develops from sensorimotor experience, that of time is more abstract, and thus relies on a mapping onto space (Boroditsky, 2000; Casasanto & Boroditsky, 2008; Clark, 1973). Accordingly, time is usually represented as sliding from left to right (in a transversal direction); this feature of time, i.e., its unidirectional sliding, allows us to conceptualize the movement of time from past (left) to future (right) as being along a transversal mental time line (MTL) (Bender & Beller, 2014; Galton, 2011). A spatial response preference has indeed been reported for stimuli that are part of an ordinal sequence, such as the months of the year (Gevers et al., 2003, 2004). Similarly, several studies have provided evidence that the mental representation of numbers is also spatialized, and is organized according to a mental number line (MNL; Restle, 1970), in which smaller numbers are placed on the left side of the MNL, whereas larger ones are placed on its right side (Hubbard et al., 2005; Umiltà et al., 2009). This association between numerical quantity and spatial position typically results in the SNARC (Spatial Numerical Association of Response Codes) effect, for which reaction times are faster for small numbers when manual responses are performed in the left side of space, and faster for large numbers when responses are performed in the right side of space (Dehaene et al., 1993). A similar effect has been observed in the temporal domain, in the so-called STEARC (Spatial–TEmporal Association of Response Codes) effect, with faster left-sided responses for stimuli with an earlier onset, and faster right-sided responses for stimuli for which onset was later (Ishihara et al., 2008). Furthermore, faster responses have been reported for past-related words and sentences requiring a left-hand response, and for future-related ones requiring a right-hand response, compared to the condition in which this matching was reversed (Santiago et al., 2007; Torralbo et al., 2006; Ulrich & Maienborn, 2010).

Although these studies support the notion that time may be represented according to a left-to-right MTL, it has been pointed out that many natural languages actually refer to time from an egocentric perspective, according to a back-to-front axis; indeed, whereas the past is usually conceived to be “behind us” (“The worst is behind us”), the future is considered to be “in front of us” (“The meeting has been moved forward”) (Boroditsky, 2011; Ulrich et al., 2012). This suggests the existence of a second type of spatial representation of time that is organized according to a back-to-front axis (Radden, 2004; Rinaldi et al., 2016; Ulrich et al., 2012). Whereas the left-to-right representation of time is probably strongly dependent on linguistic factors (such as the direction of reading and writing) (Fuhrman & Boroditsky, 2010; Ouellet et al., 2010; Tversky et al., 1991), it has been proposed that the back-to-front (sagittal) representation of time could actually be grounded in our real sensory and motor experience, namely that related to movements such as walking and running (Miles et al. 2010a). Consistent with this proposal, it has been shown that the recollection of personal events set up in the past or in the future is, respectively, associated with spontaneous backwards or forwards posture fluctuations (Miles et al., 2010a). Also, apparent backwards or forward movements in a dynamic visual display were found to be associated with the direction (future of past) of task-unrelated thoughts (Miles et al., 2010b). Further support for the hypothesis that the MTL can be represented along the sagittal axis comes from studies showing that responses to future- and past-related stimuli were faster when the response direction was congruent with the sagittal MTL (Sell & Kaschak, 2011; Ulrich et al., 2012). Finally, in a recent study, participants asked to categorize past- and future-related words through a step movement were faster and more accurate in their responses when the movement direction was compatible with the sagittal MTL, supporting the hypothesis that mentally travelling along time affects the preparation of egocentric whole-body movements (Rinaldi et al., 2016). These findings are overall consistent with proposals that sagittal representation of time could be “embodied,” or, in other words, grounded in a sensorimotor system that naturally integrates spatiotemporal information (Barsalou, 2008; Miles et al. 2010a).

Overall, this body of evidence strongly supports the existence of a sagittal MTL, and its relation to mental time travel and self-projection. However, to the best of our knowledge, no study to date has investigated the MTL in AM processes. Thus, here we aimed to test the spatiotemporal organization of autobiographical memories in their episodic and semantic components. To this purpose, we developed an experimental paradigm based on spatiotemporal interference.

## Materials and method

### Participants

Sample size was defined a priori using G*Power (Version 3.1.9.6) (Faul et al., 2007) to achieve a statistical power higher than 95%, considering an alpha of 0.05. The effect size (*η*_*p*_^*2*^ = 0.33) was derived from a previous study by Rinaldi and colleagues (Rinaldi et al., 2016). The total sample size resulting from the power analysis was 14; considering a possible dropout between the two experimental phases (~40%) and the possibility that participants failed to complete all the experimental phases (e.g., not reporting a sufficient number of memories), we finally enrolled 22 individuals. One participant did not complete the experimental task and was excluded. Thus, the final sample included 21 healthy young individuals (age range: 21–31 years; mean age: 24.810 years; SD: 2.502; 12 women).

None of the participants had a history of neurological or psychiatric disorders. All of them signed a consent form before the study began. This study was approved by the Institutional Review Board of the Department of Psychology at Sapienza University of Rome.

### Spatial compatibility task

Studies using spatiotemporal interference found that manual responses (Ulrich et al., 2012) and egocentric whole-body movements (Rinaldi et al., 2016) to past- and future-related words are faster when the response direction is compatible with a back-to-front mental timeline (i.e., forward responses for future-related words; compatible condition), rather than the opposite (i.e., backward responses for future-related words; incompatible condition) (see also *Introduction*). Based on the assumption that the past and the future are conceived as respectively behind and in front of the self, we developed a spatial compatibility task (SCT) to test the existence of the sagittal MTL in the domain of AM. The study had a 2 × 2 factorial design, with two categories and two conditions organized along the two orthogonal dimensions of memory type (episodic vs. semantic autobiographical memory – EAM and SAM, respectively) and response direction (compatible vs. not compatible with a back-to-front mental timeline, C vs. NC respectively).

#### Stimuli

Participants’ memories were collected using an adapted version of the autobiographical memory fluency task proposed by Dritschel and colleagues (Dritschel et al., 1992). For each of six possible life periods (i.e., 5–11, 11–14, 14–19, 19–24, >24 years of age, and last year) participants were asked to report as many as possible events (EAMs) and names of friends, teachers, or schoolmates, i.e., autobiographical facts (SAMs) that occurred in or corresponded to those periods. They were also asked to provide a label identifying the event or fact, without further expanding on it. It was specified, however, that events should be vivid and specific for the time and place in which they occurred. For each combination of period and category (i.e., EAM or SAM) 60 s were given. Once all periods and categories were successfully probed, participants were required to provide details about when the events occurred (for EAM) and when they met for the first time the persons they had named (for SAM). The first two items reported for each period and category were used in the SCT, assigning them randomly to the compatible and non-compatible conditions. The choice of selecting items from different epochs allowed us to be sure that, in each condition, each item clearly followed or preceded the other. Different labels were presented across conditions, to avoid spurious effects due to item repetition. However, items from the same periods were presented in different conditions, thus EAMs and SAMs were matched in terms of their age/remoteness. In each sequence, one label/item was presented for each period, as follows.

#### Procedure

With few exceptions (4/21), participants performed the autobiographical fluencies and the SCT on the same day or on two consecutive days. Either way, immediately before the administration of the SCT, the experimenter refreshed the participant’s memory of the meaning of each label, in order to avoid any misunderstanding about the correspondence between labels and memories.

Labels of the autobiographical events and facts reported during the fluency task were presented one at a time, in an unbroken sequential manner in four serially balanced sequences (one for each category and condition), in which each stimulus preceded and followed every other stimulus an equal number of times (Aguirre, 2007; Nonyane & Theobald, 2007). Each label was presented six times in a sequence, which consisted of 36 trials, with one first trial (i.e., the first trial of the sequence), five catch trials (i.e., trials in which the label was the same as the previous one), and 30 experimental trials (trials in which participants’ answers were expected based on the relation with the previous stimulus). An example of the sequences is provided in Supplementary Fig. 1 (Online Supplementary Material). Labels (font: mono, 32 pt) were presented in the center of the screen for 2,500 ms, followed by a fixation point lasting 500 ms. Screen resolution was 1,280 × 800 pixels.

**Fig. 1:**
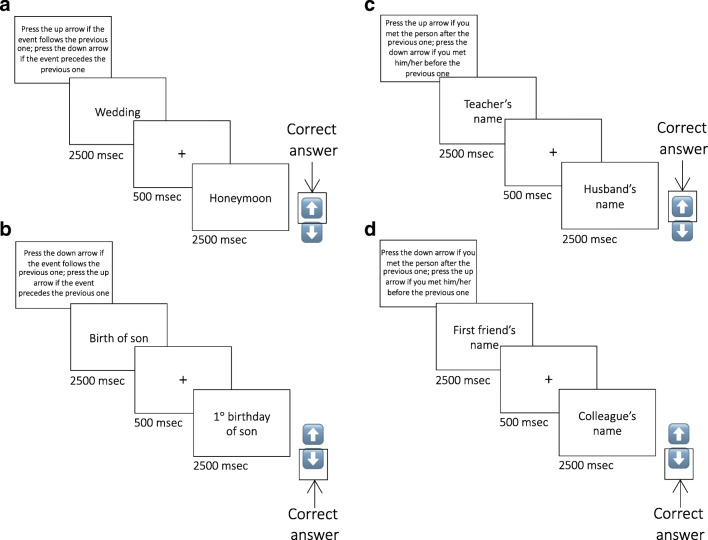
Experimental timeline and conditions. Compatible (**A**) and non-compatible (**B**) conditions of EAM are shown in the left panels. Compatible (**C**) and non-compatible (**D**) conditions of SAM are shown in the right panels. *EAM* episodic autobiographical memory, *SAM* semantic autobiographical memory

In the compatible condition (C), participants were instructed to respond as soon as possible to each stimulus, pressing the down-arrow if it preceded the previous one and the up-arrow if it followed the last one in chronological order (Fig. 1A–C). In the non-compatible condition (NC), they were required to press the down-arrow if the stimulus followed the last one (Fig. 1B–D) and the up-arrow if it preceded the last one chronologically. They were also instructed not to press any key during the first trial and during catch trials. Accuracy and response times were collected.

The presentation order of Condition (C vs. NC) and Category (SAM vs. EAM) was counterbalanced across participants. The testing phase was preceded by a practice phase, in which a set of stimuli different from that used in the testing phase was presented. The experiment was developed and administered using OpenSesame (Mathôt et al., 2012).

## Statistical analyses

For each participant, category, and condition, we calculated accuracy as the sum of correct responses during experimental trials (max. 30). We also computed the average response time (RT) for correct responses during experimental trials. Two repeated-measures ANOVAs, with Category (SAM vs. EAM) and Condition (C vs. NC) as independent variables, were performed on accuracy and RT. To further explore whether performance in the SCT reflected an underlying organization of memory, Pearson correlation coefficients between performances in the SCT and those in the autobiographical memory fluency task were computed. These analyses were run using SPSS 25. Finally, we performed a Bayesian repeated-measures ANOVA on accuracy in the SCT, to explore the possibility that the model including the interaction between the factors Category and Condition would be preferred over the model including only the main effects of the two factors. The analysis was performed using JASP (Version 0.9.2; JASP Team, 2021) setting default priors.

## Results

During the autobiographical memory fluency task, participants reported on average 38.900 autobiographical events (SD 9.534) and 65.38 autobiographical facts (SD 15.506). In the SCT, the mean number of correct responses was 26.429 in the EAM C condition (SD 2.541; the percentage of correct responses was 88%, on average), 20.524 in the EAM NC condition (SD 7.125; the percentage of correct responses was 68%, on average), 25.857 in the SAM C condition (SD 2.833; the percentage of correct responses was 86%, on average) and 23.000 in the SAM NC condition (SD 5.523; the percentage of correct responses was 77%, on average).

**Fig. 2:**
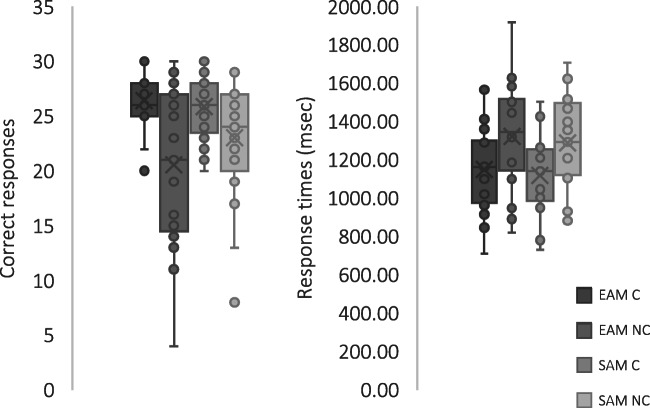
Results of the spatial compatibility task. Accuracy is shown on the left, whereas response times are shown on the right. *EAM* episodic autobiographical memory, *SAM* semantic autobiographical memory, *C* compatible, *NC* non-compatible

The repeated-measures ANOVA (sphericity: Mauchly’s W = 1.00) on accuracy revealed a main effect of Condition (*F*(1,20) = 18.137; *p* < .001; *η*_*p*_^*2*^ = 0.476): participants performed better in C than in NC (Fig. 2). Interestingly, the Category by Condition interaction was also significant (F(1,20) = 5.993; *p* = .024; *η*_*p*_^*2*^= 0.231): the difference between C and NC was higher for EAM (mean difference = 5.905; p = .001, Bonferroni’s correction for multiple comparisons was applied) than for SAM (mean difference = 2.857; *p* = 0.004, Bonferroni’s correction for multiple comparisons was applied). In other words, the NC condition worsened performance to a greater extent for EAM than for SAM (Fig. 2). The main effect of Category was not significant (*F*(1,20) = 1.272; *p* = .273; *η*_*p*_^*2*^= 0.060).

Results of the Bayesian repeated-measures ANOVA further confirmed that there was positive evidence for including the interaction between Condition and Category as a predictor of accuracy in the SCT. Indeed, the data were 1.15 times more likely under the model including both the interaction and the two main effects compared to the model with only the two main effects (results tables are available at the following link: https://osf.io/63rys/?view_only=2a1d0789513744638d2585afd8b53a2c).

The performance in the autobiographical memory fluency tasks (both episodic and semantic) correlated significantly with the proficiency in retrieving autobiographical episodic memories in the compatible condition (EAM C). Instead, no significant association was detected with the non-compatible condition of EAM (EAM NC), nor with the SAM conditions (SAM C and SAM NC). Noteworthy, EAM C was the only task condition that was not associated with other task levels. Instead, EAM NC, SAM NC, and SAM C were significantly correlated. Pearson correlation coefficients are reported in Table 1.

**Table 1:** Pearson correlation coefficients (r) and significance (one-tailed p). Significant correlations are marked in bold.

		Episodic fluency	Semantic fluency	EAM C	EAM NC	SAM C	SAM NC
Episodic fluency	r	1.000					
	p						
Semantic fluency	r	**0.451**	1.000				
	p	**0.020**					
EAM C	r	**0.417**	**0.517**	1.000			
	p	**0.030**	**0.008**				
EAM NC	r	−0.039	0.144	0.338	1.000		
	p	0.433	0.266	0.067			
SAM C	r	0.107	−0.092	0.078	**0.385**	1.000	
	p	0.322	0.346	0.368	**0.042**		
SAM NC	r	0.024	0.346	0.207	**0.615**	**0.729**	1.000
	p	0.459	0.062	0.184	**0.002**	**0.000**	

*Notes*. *EAM* = Episodic Autobiographical Memory; *SAM* = Semantic Autobiographical Memory; *C* = Compatible; *NC* = Not Compatible

With regard to RT, the repeated-measures ANOVA (sphericity: Mauchly’s W = 1.00) revealed a main effect of Condition (*F*(1,20) = 10.903; *p* = .004; *η*_*p*_^*2*^= 0.353): participants were slower in NC than in C (Fig. 2). No other significant effect was detected (Category: *F*(1,20) = 1.447; *p* = .243; *η*_*p*_^*2*^ = 0.067; Category by Condition interaction: *F*(1,20) = 0.008; *p* = .931; *η*_*p*_^*2*^ = 0.000).

### Discussion

Here we developed an experimental paradigm based on spatiotemporal interference to test the organization of episodic and semantic autobiographical memories. We found that spatiotemporal interference significantly affected performances in both EAM and SAM: individuals performed worse in the non-compatible than in the compatible condition, especially in the case of EAM. These results tie well with previous evidence coming from literature on the MTL (Rinaldi et al., 2016; Ulrich et al., 2012) and on mental time travel (Arzy et al., 2009).

Even if previous studies (e.g., Anelli et al., 2016; Arzy et al., 2009) assessed the transversal MTL asking participants to project themselves in the past or in the future, in order to decide whether events occurred earlier in the past or later in the future, this is the first study testing the sagittal MTL for autobiographical memories. Also, at odds with previous studies (e.g., Anelli et al., 2016), we only used autobiographical real-life events and facts, without including plausible future events. Thus, our results are strictly linked to the spatiotemporal organization of real autobiographical memories with interesting theoretical implications.

First, the present study provides initial evidence for the existence of a sagittal MTL for autobiographical memories. Previous studies on MTL support the idea that the past and the future are conceived, respectively, behind and in front of the ego (Núñez & Cooperrider, 2013). Thus, manual responses to past- and future-related information are usually faster when the response direction is compatible with a back-to-front MTL. Here we asked participants to continuously update their position along the MTL to decide whether the event preceded or followed the previous one, using both compatible and non-compatible back-to-front manual responses. Finding that they were more accurate and faster in the compatible than in the non-compatible condition suggests that autobiographical memories may be organized according to a sagittal MTL. These findings are overall consistent with theoretical accounts positing a strong influence of real-world sensorimotor experience on cognition in general, and specifically on memory processes (see Ianì, 2019, for a review), further suggesting that the temporal organization of autobiographical memory may also arise from this kind of sensorimotor experience.

The significant interaction we found between condition and category suggests that spatiotemporal interference is higher for EAM than for SAM, mirroring the well-known dissociation between episodic and semantic memory detected in healthy participants and brain-damaged patients. Indeed, it is consistent with evidence of a functional and neuroanatomical dissociation between episodic and semantic autobiographical memory, coming from fMRI studies on healthy participants (Levine et al., 2004). Also, patients with medial temporal lobe damage usually show episodic memory deficits with spared performance on semantic memory tasks (see, e.g., the case of K.C., described by Rosenbaum et al., 2005). Thus, it could be interesting in future studies to adopt paradigms based on spatiotemporal interference to test the spatiotemporal organization of autobiographical memory in clinical conditions characterized by memory deficits (e.g., Alzheimer disease, encephalopathies, traumatic brain injury).

Possible mechanisms explaining compatibility effects should be mentioned. As occurs for other spatial compatibility effects, such as the SNARC (Dehaene et al., 1993), it would be of interest to understand whether interference effects arise from the inherent organization of autobiographical memories, from the contrasting spatial codes in the selection of motor responses that are associated with task-relevant features, or from the temporary ad hoc adjustment triggered by the use of contrasting spatial codes (Aiello et al., 2012; Pinto, Pellegrino, Lasaponara, et al., 2019a; Pinto, Pellegrino, Marson, et al., 2019b). It has, indeed, been suggested that both the SNARC and the STEARC effect may actually arise at the stage of response selection, as a result of the conflict between a first code, based on the association between a stimulus and a response, and a contrasting one (e.g., spatial), given by the experimental instructions (Vallesi et al., 2008); when task instructions do not introduce such conflicting coding, conversely, compatibility effects may disappear (Anelli et al., 2018). Also, previous studies on sentence processing suggested that spatiotemporal compatibility effects may be non-automatic, since they are abolished when the task does not explicitly require to give a temporal judgment, and temporal information is thus not task-relevant (Ulrich et al., 2012; Ulrich & Maienborn, 2010). Present findings support the hypothesis that a pre-experimental link, shaped by sensorimotor experience, exists between episodic autobiographical memories and spatial codes, such as that performing judgments on the sequence of one’s episodic autobiographical events is facilitated when temporal information can be mapped consistently with this spatial code. Further studies, however, will be needed to establish whether the sagittal MTL is automatically activated when recollecting episodic autobiographical memories, or its activation requires the explicit processing of temporal information related to the order of events.

It is also important to point out that possible alternative explanations for the present findings should be taken into account. In this respect, it has been shown that effects such as the SNARC do not uniquely arise from a visuospatial representation of the mapped dimension, but can also be triggered by verbal associations between such dimensions and specific labels (e.g., between small/large numbers and labels such as “left”/”right”; Gevers et al., 2010). One could thus hypothesize that the effect we reported here is simply related to a conflict arising between concepts and motor responses. Crucially, if this were the case, we should observe that spatial compatibility indiscriminately affects semantic and episodic autobiographical memory. However, having observed that spatial compatibility affected episodic autobiographical memory to a greater extent than semantic autobiographical memory, we have to dismiss such an explanation, at least for the episodic component. Thus, a more conservative interpretation of the present results is that episodic autobiographical memories (and not semantic ones) are mapped along a sagittal MTL. This interpretation is also consistent with evidence from similar paradigms assessing the transversal (Ulrich & Maienborn, 2010) and sagittal (Ulrich et al., 2012) MTL. Furthermore, it is in line with the notion that episodic autobiographical memory encompasses events with a unique spatiotemporal context, whereas semantic autobiographical memory corresponds to memory for information with no clear temporal and spatial context. Once again, this interpretation fits well with neuropsychological evidence of the dissociation between episodic and semantic memory mentioned above. Interestingly, the pattern of correlations we detected between performances in the SCT and in the autobiographical memory fluency task is also in line with a segregation between EAM and SAM, and with the possibility that EAMs are organized according to a back-to-front MTL.

It is also worth noting that, although in most languages the conceptualization of past and future as, respectively, behind and in front of us is consistent with the sensorimotor experience related to walking and running, exceptions do exist. A well-known example is the case of Aymara speakers, who conceive the future and the past as being respectively “in back” and “in front” of the ego (Núñez & Sweetser, 2006). Thus, an important issue is whether the representation of EAMs along a sagittal MTL is a universal phenomenon, consistent across different cultures. It has been suggested that the sagittal MTL originates from at least two types of sources, namely the sensorimotor experience associated with walking, and the metaphoric mapping between time and space in language (Ding et al., 2020). Although results of the present study are consistent with previous evidence of a sagittal MTL in western populations (Miles, Karpinska, et al., 2010b; Miles, Nind, & Macrae, 2010a; Rinaldi et al., 2016), future studies should address whether the organization of EAM along a sagittal MTL shows cultural variations related to differences in metaphoric mapping habits between space and time.

Also, considering that the sagittal MTL likely develops from one's own bodily experiences, namely moving forward leaving everything behind us, it could be of interest to test spatiotemporal interference for autobiographical memory in more ecological setups, with egocentric whole-body movements. Finally, it is possible that the temporal distance between events may also affect the spatial compatibility effect. Here, we included only events/facts participants clearly remembered (i.e., the first two items provided during the autobiographical fluency task). Thus, only one label was presented in each serially balanced sequence/condition. This choice allowed us to define events as occurring before or after each one, unequivocally; however, it inevitably reduced the variability in the distance between events and did not allow modulating parametrically the distance between such events. Future studies should test whether the distance between events significantly affects the spatial compatibility effect we reported here for episodic autobiographical memory. Also, future studies should test the impact of the timescale on spatiotemporal interference, investigating possible similarities between different scales (Moreton & Ward, 2010), and also attempting to disentangle how fine-grained the temporal coding of autobiographical memories is, and its possible interaction with memory age (Boccia et al., 2019).

## Supplementary Information

ESM 1 (DOCX 14 kb)

ESM 2 (DOCX 34 kb)
