# Supplementary material for: Mental representation of autobiographical memories along the sagittal mental timeline: Evidence from spatiotemporal interference
Source: Psychon Bull Rev. 2021 Mar 29;28(4):1327–35. doi: 10.3758/s13423-021-01906-z (PMC8367924; doi:10.3758/s13423-021-01906-z)
Supplement: Supplementary file 1 — (DOCX 14 kb) [file 13423_2021_1906_MOESM1_ESM.docx]

|  |  | Episodic fluency | Semantic fluency | EAM C | EAM NC | SAM C | SAM NC |
| --- | --- | --- | --- | --- | --- | --- | --- |
| Episodic fluency | r | 1.000 |  |  |  |  |  |
|  | p |  |  |  |  |  |  |
| Semantic fluency | r | **0.451** | 1.000 |  |  |  |  |
|  | p | **0.020** |  |  |  |  |  |
| EAM C | r | **0.417** | **0.517** | 1.000 |  |  |  |
|  | p | **0.030** | **0.008** |  |  |  |  |
| EAM NC | r | -0.039 | 0.144 | 0.338 | 1.000 |  |  |
|  | p | 0.433 | 0.266 | 0.067 |  |  |  |
| SAM C | r | 0.107 | -0.092 | 0.078 | **0.385** | 1.000 |  |
|  | p | 0.322 | 0.346 | 0.368 | **0.042** |  |  |
| SAM NC | r | 0.024 | 0.346 | 0.207 | **0.615** | **0.729** | 1.000 |
|  | p | 0.459 | 0.062 | 0.184 | **0.002** | **0.000** |  |

**Table 1** Pearson correlation coefficients (r) and significance (one-tailed p). Significant correlations are marked in bold. *Notes*. EAM = Episodic Autobiographical Memory; SAM = Semantic Autobiographical Memory; C = Compatible; NC = Not Compatible.
