# Supplementary material for: Mental representation of autobiographical memories along the sagittal mental timeline: Evidence from spatiotemporal interference
Source: Psychon Bull Rev. 2021 Mar 29;28(4):1327–35. doi: 10.3758/s13423-021-01906-z (PMC8367924; doi:10.3758/s13423-021-01906-z)
Supplement: Supplementary file 2 — (DOCX 34 kb) [file 13423_2021_1906_MOESM2_ESM.docx]

**Supplementary Figure 1** – An example of the sequences used in the present study, in which the first trial, catch trials and experimental trials are highlighted. Each label (event/fact) is associated uniquely with a specific number.
